# Anti-*Listeria* activity of *Lactococcus lactis* subsp. *lactis* LAB3 cells entrapped in alginate beads: effects of inoculum size, alginate bead formulation, and atmosphere composition

**DOI:** 10.3389/fmicb.2025.1663475

**Published:** 2025-08-21

**Authors:** Yan He, Pascal Degraeve, Nadia Oulahal

**Affiliations:** BioDyMIA Research Unit, Université de Lyon, Université Claude Bernard Lyon 1, ISARA Lyon, Bourg-en-Bresse, France

**Keywords:** bioprotective lactic acid bacteria, sodium alginate, sodium caseinate, anti-*Listeria* activity, food preservation, modified atmosphere packaging

## Abstract

Bioprotective *Lactococcus lactis* LAB3 cells that produce bacteriocin-like substances were entrapped in 4% (w/w) sodium alginate matrices, either with or without 10% (w/w) sodium caseinate. The effects of bead formulation—alginate alone or combined with caseinate, with or without the addition of 20% (w/w) MRS broth or M17 broth—on the culturability of *Lc. Lactis* LAB3 cells within the beads and their anti*-Listeria* activity were assessed over 12 days of storage at 30 °C in closed bottles. Calcium–alginate–caseinate beads supplemented with MRS broth proved most effective in preserving both culturability and anti-*Listeria* activity. Inoculum size (~10^6^ or ~10^8^ CFU mL^−1^ initially) also played a role: only the higher initial inoculum yielded significant anti-*Listeria* activity after 12 days at 30 °C, despite a gradual decline over time, likely due to rapid nutrient depletion. In order to evaluate the feasibility of combining modified-atmosphere packaging with the addition of the *Lc. lactis* LAB3 bioprotective strain for preserving perishable foods, a prerequisite was to evaluate whether anti-*Listeria* activity persisted after 4 days of storage at 30 °C in calcium–alginate–caseinate beads containing either MRS or M17 broth under three different atmospheres. Beads entrapping *Lc. lactis* LAB3 cells stored in 20% (v/v) O_2_–80% (v/v) N_2_ or in 60% (v/v) O_2_–40% (v/v) N_2_ retained their anti-*Listeria* activity, whereas storage in 20% (v/v) CO_2_–80% (v/v) N_2_ impaired this activity.

## Graphical abstract

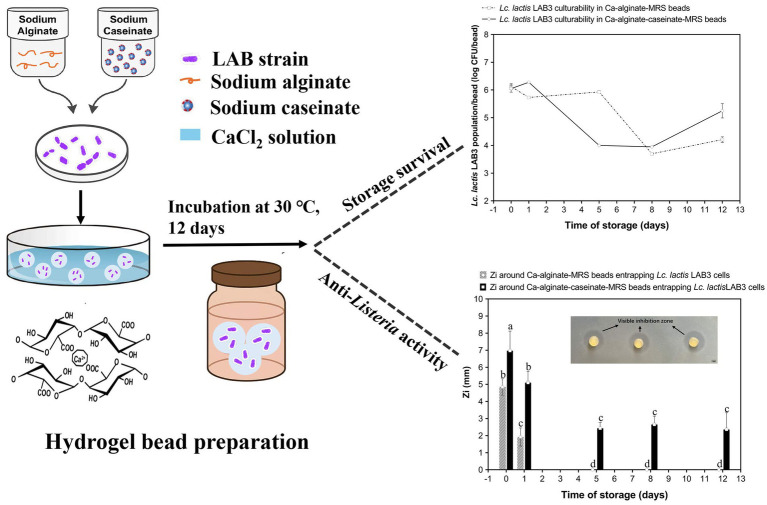

## Highlights

- Anti-*Listeria* activity and culturability of *Lactococcus lactis* LAB3 entrapped in alginate beads were evaluated.
- MRS broth effectively maintained the culturability and anti-*Listeria* activity of *Lc. lactis* LAB3 cells over a 12-day period at 30 °C.
- A higher inoculum size of cells resulted in promoted and prolonged anti-*Listeria* activity.
- Anti-*Listeria* activity of *Lc. lactis* LAB3 was preserved in 20% (v/v) O_2_ − 80% (v/v) N_2_ and 60% (v/v) O_2_ − 40% (v/v) N2 atmospheres.

## 1 Introduction

Listeriosis, caused by the pathogenic bacterium *Listeria monocytogenes*, remains a significant public health concern and is often associated with the consumption of contaminated food. A report covering 30 EU/EEA countries revealed that between 2018 and 2023, an average of 100–400 confirmed listeriosis cases per 100,000 population occurred monthly, with a pronounced peak during the summer months (European Centre for Disease Prevention and Control, 2024). This seasonal surge underscores the pressing need for effective interventions to prevent *Listeria* contamination. The persistence of *L. monocytogenes* in food environments is largely due to its remarkable adaptability. It can survive across a broad pH range (4.6–9.5), tolerate high salt concentrations (up to 20%), and grow within a temperature range of −0.4 °C to 45 °C (Osek et al., 2022). These characteristics make controlling this pathogen particularly challenging, especially in ready-to-eat products such as processed meats and dairy products, which are frequently implicated in listeriosis outbreaks (FAO and WHO, 2022).

To address these challenges, there is a growing interest in sustainable, natural food preservation methods. One promising approach involves the use of active edible coatings or films made from polysaccharides or proteins, which can incorporate functional ingredients such as antimicrobial compounds (e.g., organic acids, bacteriocins, and essential oils) (Cazón et al., 2024; Sabbir et al., 2025; Tilwani et al., 2025) to inhibit the growth of pathogenic microorganisms. Another strategy is the incorporation of live bacteriocin-producing lactic acid bacteria (LAB) directly into polymer matrices, which has shown potential as a bioprotective measure (Sánchez-González et al., 2014; Silva et al., 2022; Fernandes et al., 2025). LAB exhibit superior antibacterial activity compared to single antimicrobial agents or chemical preservatives, owing to their ability to produce a diverse range of bioactive metabolites, including organic acids, short-chain fatty acids, carbohydrates, antimicrobial peptides (notably bacteriocins), enzymes, vitamins, cofactors, immune-signaling compounds, hydrogen peroxide, reuterin, and diacetyl (Moradi et al., 2020; Haryani et al., 2024; Zhang et al., 2024; Kuhn et al., 2025). Their antimicrobial action against spoilage microorganisms and pathogens on food surfaces also involves competition for space or nutrients (Silva et al., 2018). More importantly, the Generally Recognized as Safe (GRAS) status granted by the U.S. Food and Drug Administration (FDA) to most LAB supports their inclusion in edible coatings and films, aligning with consumer demand for preservative-free, safe, and natural food products.

However, incorporating live LAB cells into biopolymeric matrices still presents notable challenges. A primary concern is ensuring the prolonged maintenance of microbial viability, as reduced viability during storage compromises the LAB’s antimicrobial efficacy. Additionally, the viability and antagonistic activity of LAB are highly susceptible to adverse environmental conditions (including temperature fluctuations, pH extremes, and moisture changes). In previous studies (Léonard et al., 2014; Léonard et al., 2015), we reported that *Lc. lactis* subsp. *lactis* LAB3 cells entrapped in liquid alginate–caseinate two-phase systems supplemented with MRS broth exhibited higher culturability as well as anti-*Listeria* activity than when entrapped in the same systems with only alginate. Building on previous findings, this study aimed to develop an optimized hydrogel bead system for the entrapment of *Lc. lactis* subsp. *lactis* LAB3 by examining how inoculum level, bead composition (with or without caseinate and culture media), and modified atmosphere conditions affect its long-term culturability and anti-*Listeria* performance.

Given that M17 broth is typically used for lactococci cultivation, while MRS broth is preferred for lactobacilli, the viability and anti-*Listeria* activity of *Lc. lactis* susp. *Lactis* LAB3 in alginate–caseinate beads and alginate beads supplemented with either M17 broth or MRS broth were monitored. Beads were initially inoculated with 10^6^ or 10^8^ CFU mL^−1^ of *Lc. lactis* susp. *Lactis* LAB3 cells. Culturability and anti-*Listeria* activity were monitored over 12 days at 30 °C to simulate a cold chain break.

Despite the promises of the use of bioprotective LAB, such as *Lc. lactis* subsp. *lactis* LAB3, modified atmosphere packaging (MAP) is the preferred technology commonly used to extend the shelf-life and/or improve the microbial safety of perishable foods (Habiba et al., 2025). Oxygen (O_2_), carbon dioxide (CO_2_), and nitrogen (N_2_) are the most common gases used in MAP. Carbon dioxide is soluble both in water and in fat and has a bacteriostatic effect (Moun et al., 2025). Oxygen inhibits strictly anaerobic bacteria and enhances the growth of aerobic microorganisms. High oxygen MAP (typically 60–80% (v/v) (O₂)) is also used to maintain the bright red color of meat due to oxymyoglobin (Corlett et al., 2024). Indeed, elevated O₂ levels inhibit metmyoglobin formation on the meat surface without significantly accelerating the growth of aerobic microorganisms (Djenane et al., 2005). N₂ acts as an inert filler gas with no antimicrobial activity, serving primarily to balance the gas composition. Consistent with the principles of hurdle technology, bioprotective LAB applications are particularly promising when combined with other preservation strategies, such as modified atmosphere packaging (MAP), to extend shelf life and/or improve the microbial safety of perishable foods. Accordingly, the ability of *Lc. lactis* subsp. *lactis* LAB3 cells, entrapped in alginate–caseinate systems containing either MRS broth or M17 broth, to maintain culturability and anti-*Listeria* activity was compared under three conditions: 20% O_2_–80% N_2_ (mimicking air composition), in a 20% CO_2_–80% N_2,_ and 60% O_2_–40% N_2_ (mimicking high-oxygen modified atmosphere packaging). This comparison aimed to identify which atmosphere composition can be effectively combined with entrapped bioprotective *Lc. lactis* subsp. *lactis* cells to extend shelf life and/or control *L. monocytogenes* growth in perishable foods.

## 2 Materials and methods

### 2.1 Bacterial strain and culture conditions

All experimental procedures, including strain storage and maintenance, medium preparation, and antimicrobial activity assays, were performed in the microbiology laboratory of the BioDyMIA Research Unit (Université Lyon 1, France). The *Lc. lactis* subsp. *lactis* LAB3 strain, used in this study for its bioprotective properties, is a commercial dairy starter (MD089, Ezal line, Rhône Poulenc, Dangé Saint-Romain, France) (Lamboley et al., 1997). This strain is known for its lactic acid production and its ability to produce bacteriocin-like inhibitory substances active against *Listeria* spp. *Lc. lactis* subsp. *lactis* LAB3 was stored at −20 °C in de Man Rogosa and Sharpe (MRS) broth (Biokar Diagnostics, Beauvais, France) supplemented with 15% (v/v) glycerol for long-term preservation. Before experimentation, *Lc. lactis* cells were recovered by subculturing in anaerobic conditions in MRS broth (pH 6.1, after autoclaving). The initial subculture was prepared by inoculating 10% (v/v) of the stored culture into MRS broth and incubating it at 30 °C for 24 h without shaking in an oven. This was followed by a secondary subculture, where the culture was transferred into fresh MRS broth and incubated under the same conditions for 14–16 h, reaching the late exponential growth phase. All operations were performed under sterile conditions.

The target strain, *Listeria innocua* ATCC 33090 (a surrogate strain for the pathogenic strain *L. monocytogenes*), was obtained from stock cultures with 15% (v/v) glycerol stored at −40 °C. *L. innocua* ATCC 33090 was subcultured twice in Tryptone Soy Broth (TSB, Biokar Diagnostics, Beauvais, France) under sterile conditions. The first subculture was performed by inoculating 10% (v/v) of the stock culture into fresh TSB and incubating at 30 °C aerobically without shaking for 8 h. A second subculture was then prepared by inoculating 10% (v/v) into fresh TSB and incubating at 30 °C aerobically for 14–15 h. Fresh cultures for experiments were obtained by inoculating 20% (v/v) into fresh TSB, followed by incubation under the same conditions for 4–5 h to reach the exponential growth phase, indicated by an optical density at 600 nm (OD600) of approximately 0.5. OD600 measurements were performed using a Jenway 6,300 spectrophotometer (Felsted, UK) to estimate cell density.

### 2.2 Preparation of gelled polymeric matrices with bacterial cells

#### 2.2.1 Incorporation of bacteria in biopolymeric matrices

To prepare a 4% (w/w) sodium alginate dispersion, 4 g of sodium alginate (Sigma-Aldrich, Darmstadt, Germany) was dissolved in 96 mL of sterile deionized water under sterile conditions. The solution was stirred overnight using a magnetic stirrer (RH basic 2 IKA™, Staufen im Breisgau, Germany) to ensure complete dissolution. A 10% (w/w) sodium caseinate (Sigma-Aldrich, Darmstadt, Germany) dispersion was prepared by dissolving sodium caseinate in sterile deionized water through continuous stirring with a magnetic stirrer. Both dispersions were centrifuged at 12,500 × g for 15 min at 20 °C to remove any undissolved particles. The pH of the solutions was then adjusted to 7.0.

*Lc. lactis* LAB3 cells were harvested by centrifugation at 5,000 × g for 15 min at 4 °C from a culture in MRS broth [approximately 10^10^ colony-forming units per milliliter (CFU mL^−1^)]. The culture medium was discarded, and the cell pellet was washed twice with Tryptone Salt broth (TS) (Biokar Diagnostics, Beauvais, France) to remove residual medium. The cells were then resuspended in fresh MRS broth, M17 broth, or sterile deionized water at a final concentration of 10^~8^ CFU mL^−1^ to produce beads containing MRS broth, M17 broth, or no extra media. The compositions of MRS broth and M17 broth are shown in Table 1. Two types of biopolymeric beads were prepared for bacterial entrapment: calcium–alginate–caseinate beads composed of 1.5% (w/w) sodium alginate and 4% (w/w) sodium caseinate, and calcium–alginate beads composed of 1.5% (w/w) sodium alginate only. For each bead type, three formulations were prepared by incorporating either no culture medium, 20% (w/w) MRS broth, or 20% (w/w) M17 broth. In all formulations, a bacterial suspension was added at 20% (w/w) to the polymeric mixtures to produce the final biopolymeric matrices for bead preparation. Control beads were prepared using the same biopolymeric formulations but without the addition of *Lc. lactis* LAB3 cells.

**Table 1:** MRS broth and M17 broth compositions.

Component	MRS broth (pH 6.1 ± 0.2 at 25 °C) (g/L in deionized water)	M17 broth (pH 5.8 ± 0.2 at 25 °C) (g/L in deionized water)
Peptones	20.00	–
Tryptone	–	2.50
Peptic digest of meat	–	2.50
Papaic digest of soybean meal	–	5.00
Autolytic yeast extract	5.00	–
Yeast extract	–	2.50
Meat extract	–	5.00
Glucose	20.00	–
Lactose	–	5.00
Tween 80	1.08	–
Dipotassium phosphate	2.00	–
Sodium acetate	5.00	–
Sodium glycerophosphate	–	19.00
Ammonium citrate	2.00	–
Magnesium sulfate	0.20	0.25
Manganese sulfate	0.05	–
Ascorbic acid	–	0.25

#### 2.2.2 Preparation of alginate-based hydrogel beads

The preparation of alginate–caseinate hydrogel beads was conducted according to the previous method of Ding et al. (2022) with some modifications. All steps were carried out in a sterile environment. A 30 mL volume of calcium chloride (0.1 mol·L^−1^) was placed in a 90 mm diameter Petri dish, and 50 μL of the sample solution was evenly dispensed using a 1-mL syringe (18G, Terumo, Tokyo, Japan), resulting in beads with a diameter of approximately 2–3 mm, as shown in Table 2. The beads were soaked in the calcium solution for at least 15 min, then rinsed and washed with 10 mL of sterile water. The diameter of at least 15 beads was measured using a micrometer gauge to ensure accuracy. Beads of uniform size and shape were collected using sterile forceps into a sterile bottle and marked as day 0 (D0). The hydrogel beads were incubated in sealed bottles at 30 °C or used in further experiments. Cell counts and antimicrobial activity were assessed on day 0 (D0), day 1 (D1), day 5 (D5), day 8 (D8), and day 12 (D12).

**Table 2:** Initial inoculum load of *Lactococcus lactis* LAB3 in beads and the diameters of the beads.

Group	Bead	Diameter of bead (mm), *n* = 15	Initial inoculum load (log CFU/bead), *n* = 3
a	Calcium–alginate without media	2.84 ± 0.04^a^	5.18 ± 0.13^c^
	Calcium–alginate–caseinate without media	2.96 ± 0.11^a^	6.63 ± 0.20^a^
b	Calcium–alginate-MRS	2.89 ± 0.08^a^	6.12 ± 0.11^b^
	Calcium–alginate–caseinate-MRS	2.80 ± 0.08^a^	6.05 ± 0.13^b^
c	Calcium–alginate-M17	2.89 ± 0.02^a^	6.09 ± 0.03^b^
	Calcium–alginate–caseinate-M17	2.88 ± 0.10^a^	5.92 ± 0.11^b^

Means in the same column followed by the same superscript letter are not significantly different (*p* > 0.05) according to Tukey’s post hoc test.

#### 2.2.3 Enumeration of bacteria during storage

For cell counting, alginate-based beads stored at 30 °C were analyzed on D0, D1, D5, D8, and D12. Cell counts were determined using classical enumeration on agar. Specifically, a single bead from each time point and storage condition was dissolved in 10 mL of sodium phosphate buffer (0.1 mol/L, pH 7.0) at room temperature for approximately 5 h. Following dissolution, serial dilutions were prepared using the TS medium. From each dilution, 10 μL aliquots were spread onto solid MRS agar (Biokar Diagnostics, Beauvais, France). The plates were then incubated at 30 °C for 48 h under aerobic conditions in an oven. After incubation, CFUs were counted to determine cell culturability within the beads. At least three replicates were performed. Although analyzing a single bead per time point may limit statistical robustness, preliminary tests showed minimal variation between beads, making this approach reasonably representative for this study.

#### 2.2.4 Antagonistic activity assay

To assess the anti-*Listeria* activity of hydrogel beads stored at 30 °C for 0, 2, 5, 8, and 12 days, a fresh *Listeria innocua* ATCC 33090 culture was diluted in TS medium to a concentration of 10^7^ CFU mL^−1^. The *L. innocua* ATCC 33090 culture was then mixed with melted warm Tryptone Soy Agar (TSA) (Biokar Diagnostics, Beauvais, France) at 45 °C at a ratio of 5% (v/v) to obtain a final concentration of 5 × 10^5^ CFU mL^−1^. Approximately 30 mL of the TSA-*Listeria* mixture was poured into 140 mm diameter Petri dishes, and after solidification, hydrogel beads were placed on the agar surface using sterile forceps. Another 20 mL of TSA containing *L. innocua* ATCC 33090 was then poured over the solidified layer. To ensure proper diffusion of antibacterial substances, including nisin, the plates were first incubated at 4 °C for 3 h. Subsequently, the plates were incubated at 30 °C for 48 h under aerobic conditions.

Beads without LAB3 cells were used as negative controls. The presence of a clear inhibition zone around the beads indicated positive antagonistic activity. These control beads were prepared using the same polymeric matrix compositions but without the addition of LAB3 cells. When tested for antimicrobial activity against *L. innocua* ATCC 33090, no inhibition zones were observed, confirming the absence of intrinsic antibacterial effects from the biopolymeric matrix itself. The diameter of the inhibition zone (Zi) was measured with a micrometer screw and calculated using the formula:


$$\begin{array}{l} \mathrm{Zi}\;(\mathrm{mm})=\text{Diameter of inhibition zone observed}\;(\mathrm{mm})-\\ \text{Diameter of beads}\;(\mathrm{mm}) \end{array}$$

#### 2.2.5 Modified atmosphere storage conditions

Calcium–alginate–caseinate beads containing MRS broth (termed Ca-alg-cas-MRS) or M17 broth (termed Ca-alg-cas-M17), with entrapped LAB3 cells, were incubated with TSA-*L. innocua* ATCC 33090 at 30 °C for 4 days under three modified atmosphere conditions: 20% O_2_ + 80% N_2_ (mimicking air composition), 20% CO_2_ + 80% N_2_ (mimicking carbon dioxide–modified atmosphere), and 60% O_2_ + 40% N_2_ (mimicking high-oxygen modified atmosphere), in closed cylinders. The diameters of beads were 2.79 ± 0.07 mm and 2.79 ± 0.10 mm, respectively (*n* = 15). The initial inoculum load of *Lc. lactis* LAB3 in the liquid matrices before bead formation was approximately ~10^9^ CFU/mL. The experiment was performed in cylinders by placing corresponding samples deposited onto TSA inoculated with *L. innocua* ATCC 33090 at a final concentration of 5 × 10^5^ CFU mL^−1^. The cylinders were then sealed under the designated gas mixture supplied by Air Liquide France Industrie (Paris, France). They were immediately placed in a refrigerator for 3 h at 4 °C before being transferred to an oven at 30 °C. The oxygen content (oxygen sensor point; SP-PSt3-NAU-D5-YOP, PreSens GmbH, Regensburg, Germany) was observed during storage. During the 4-day storage, the oxygen content in the controlled atmosphere storage remained unchanged or slightly decreased, but the change never exceeded 10% of the original oxygen content. It was verified that no inhibition zones were observed around control beads without LAB3 cells. Antimicrobial activity was quantified by measuring the Zi value using the agar diffusion method as described in *2.2.4.*

## 3 Statistical analysis

The experiment was repeated three times for each group, and the experimental data were expressed as mean ± standard deviation. Statistical analysis was performed using analysis of variance (ANOVA) with SPSS software (IBM SPSS Statistics 26 version). The data were ranked, and statistical differences were identified using a one-way ANOVA test along with either the least significant difference (LSD) *post hoc* test or Tukey post hoc test.

## 4 Results and discussion

### 4.1 Culturability of *Lactococcus lactis* LAB3 entrapped in calcium alginate-based beads supplemented or not with different microbiological culture media

**Figure 1:**
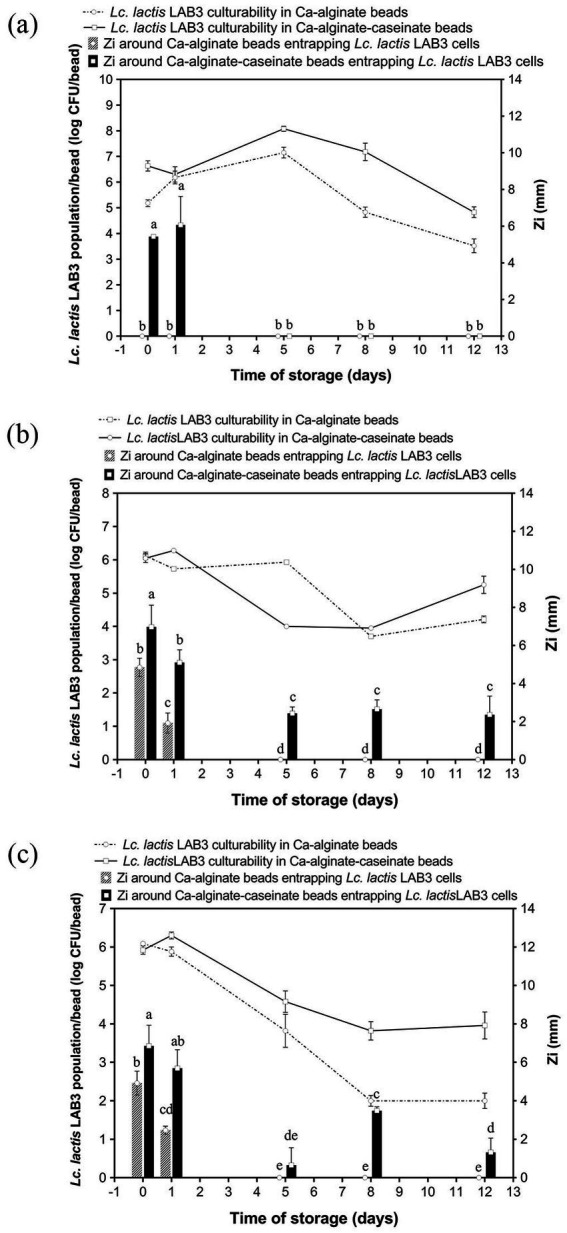
Anti-*Listeria*, activity and *Lactococcus lactis* LAB3 population in Ca-alginate beads and in Ca-alginate-caseinate beads over 12 days storage at 30 °C in air. Biopolymeric matrices compositions of beads: Ca-alginate bead: 1.5% (w/w) sodium alginate; Ca-alginate-caseinate bead: 1.5% (w/w) sodium alginate, 4% (w/w) sodium caseinate. **(a)** no microbiological culture medium **(b)** 20% (w/w) MRS broth, and **(c)** 20% (w/w) M17 broth, was included in each bead. The cell counts reduction and anti-*Listeria* activity were monitored over 12 days storage at 30 °C. All beads were incubated under sealed bottles without modified gas mixtures. Initial inoculum load of *Lc. lactis* LAB cells in initial liquid biopolymeric matrices: ~10^8^ CFU mL^−1^. Cell counts reduction in Ca-alginate-cascinate bead and Ca-alginate bead were detected and displayed in log CFU/bead (colony-forming units). The solid line represents counts in Ca-alginate-caseinate beads, and dotted line for Ca-alginate beads, determined using classical enumeration on agar. The antibacterial activity was estimated by measuring the diameters of *Listeria innocua* 33,090 growth inhibition zones around Ca-alginate-caseinate bead or Ca-alginate bead placed on the surface of agar plates. The inoculum concentration of *L. innocua* 33,090 used for the antimicrobial activity (Zi) assay was 5 × 10^5^ CFU mL^−1^. No inhibition zone was observed in Ca-alginate-cascinate bead and Ca-alginate bead without *Lc. lactis* LAB3 entrapped. Data represents the mean ± standard deviation (SD) of three independent experiments. Different letters above bars indicate significant differences (*p* < = 0.05, *n* = 1) in each figure.

To determine whether MRS broth or M17 broth components affect the growth and survival of LAB3 cells entrapped in alginate-based hydrogel beads, Ca–alginate–caseinate beads, and calcium–alginate beads containing no microbiological culture media (control) (Figure 1a), MRS broth (Figure 1b), and M17 broth (Figure 1c), were initially loaded with LAB3 cells at ~10^6^ CFU/bead (Table 2). The average diameter of the beads in each group was measured to be 2.90 mm, ranging from 2.84 mm to 2.96 mm, and there was no statistical difference between the groups (*p* > 0.05) (Table 2). The culturability of LAB3 cells entrapped in each of these beads was monitored over 12 days of storage at 30 °C: it was determined using classical enumeration on agar. Notably, the highest *Lc. lactis* LAB3 cell viability after 12 days of incubation at 30 °C in air (i.e., 5.25 ± 0.26 log CFU/bead) was obtained with alginate–caseinate beads supplemented with MRS broth.

The LAB3 cell population reached its maximum after 5 days at 30 °C (Figure 1a), with 7.15 ± 0.21 log CFU/bead observed for cells entrapped in alginate beads, and a higher cell population (i.e., 8.08 ± 0.10 log CFU/bead) when entrapped in alginate–caseinate beads, despite the absence of culture medium within the beads. This increase may be attributed to an initial osmotic effect that favors bacterial growth. Bacteria are known to possess complex stress-sensing systems and defense mechanisms that enable them to survive under harsh conditions and adapt to sudden environmental changes (Sunny-Roberts and Knorr, 2007). These stress responses are often accompanied by the transient induction of both specific and non-specific proteins, along with physiological adaptations, which collectively enhance bacterial resistance to more severe environmental challenges. The positive effect of proteins such as caseinate on *Lc. lactis* survival during storage is consistent with the findings of Settier-Ramírez et al. (2019), who reported that the presence of proteins or protein hydrolysates in a PVOH matrix used to embed *Lc. lactis* cells created a more favorable environment for bacterial survival. Similarly, Pinilla et al. (2025) observed that the addition of a soluble protein source to a whey-based culture medium promoted LAB growth. Casein can serve as a nitrogen source, providing essential amino acids for cell growth, which may explain the positive effect of caseinate addition on *Lc. lactis* LAB3 culturability observed in the present study (Savijoki et al., 2006). Moreover, alginate and caseinate are not miscible under the conditions used (1.5% (w/w) sodium alginate and 4% (w/w) sodium caseinate at an initial pH value of 7.0) (Léonard et al., 2013). In such alginate–caseinate aqueous two-phase systems, *Lc. lactis* LAB3 cells have been shown to localize exclusively in the caseinate phase, thereby residing in a different environment from the alginate phase. However, *Lc. lactis* LAB3 cell counts decreased significantly after day 5 at 30 °C, decreasing by 3.63 + 0.27 log and 3.25 + 0.21 log on day 12 compared with day 5 alginate and alginate–caseinate beads, respectively.

*Lc. lactis* LAB3 population development in alginate beads and alginate–caseinate beads, both supplemented with MRS broth over 12 days of storage at 30 °C in air, is shown in Figure 1b. Surprisingly, despite the addition of MRS broth, the *Lc. lactis* LAB3 cell population did not increase during the first days, unlike in the absence of microbiological culture medium. However, interestingly, the decrease in the *Lc. lactis* LAB3 population was more limited in alginate–caseinate beads than in alginate beads, with a 5.25 ± 0.26 log CFU/bead population after 12 days at 30 °C. Since MRS broth contains 20 g/L glucose, which can be converted to lactic acid by *Lc. lactis* LAB3, this positive effect of caseinate might also be due to its high buffering capacity, which limits pH decrease and thus acidic stress for *Lc. lactis* LAB3 cells (Léonard et al., 2014).

Finally, the evolution of *Lc. lactis* LAB3 cell populations in alginate beads and in alginate–caseinate beads supplemented with M17 broth over 12 days of storage at 30 °C in closed bottles is shown in Figure 1c. Surprisingly, while a similar decrease in *Lc. lactis* LAB3 cell populations in alginate–caseinate beads was observed over 12 days at 30 °C when the beads were supplemented with either M17 broth or MRS broth, a greater decrease in *Lc. lactis* LAB3 populations was noted when alginate beads were supplemented with M17 broth, contrary to what was observed when alginate beads were supplemented with MRS broth (Figure 1b). This difference is likely due to compositional differences between MRS broth and M17 broth (Table 1). However, further studies are needed to identify which specific components of M17 broth and MRS broth are responsible. According to the literature, MRS is ideal for cultivating *Lactobacillus*, while M17 is more suitable for *Lactococcus* and *Streptococcus* (Oğuz and Andiç, 2024). Süle et al. (2014) have demonstrated that there may be a significant variation in the growth of different strains in MRS and M17 media. MRS medium is optimal for LAB due to its glucose content and nutrient-rich composition, supporting robust growth and viability. However, sometimes MRS medium does not sufficiently satisfy the nutritional requirements for all LAB strains. Ko et al. (2024) optimized MRS medium by incorporating glucose, sucrose, and mannose (6.7 g/L each) as carbon sources, replacing beef extract with soy peptone (19.11 g/L) as a plant-based nitrogen source, and adding tomato juice (1 g/L) and L-ascorbic acid (0.1 g/L) as growth factors. They concluded that the optimized MRS showed higher growth rates for most psychrotrophic kimchi LAB strains. In the present study, the highest *Lc. lactis* LAB3 population after 12 days of storage at 30 °C (i.e., 5.25 ± 0.26 log CFU/bead) was obtained for *Lc. lactis* LAB3 cells embedded in alginate–caseinate beads supplemented with MRS broth.

Silva et al. (2022) recently reported that the viability of two LAB strains incorporated in an alginate-maltodextrin-glycerol matrix maintained high numbers for 10 days of storage at 4 °C and 10 °C. The substrate provided by the coating matrix without any nutrient medium was adequate to maintain the survival of both strains for 10 days at refrigeration temperatures. The high viability of the LAB strains at refrigeration temperatures could be due to a decrease in bacterial metabolic activity, as well as in enzymatic and chemical activities (Ghayoomi et al., 2025). However, these findings are not consistent with reports from other authors, who observed that films formulated with methylcellulose and sodium caseinate, incorporating *Lb. acidophilus* or *Lb. reuteri,* resulted in a 3-log CFU/cm^2^ reduction of LAB after 5 days of storage at 5 °C (Sánchez-González et al., 2014). This difference might result from differences of tolerance to low temperature from one LAB strain to another one: indeed, the optimal growth temperature for the majority of LAB strains ranges from 30 °C to 45 °C and temperatures far below this temperature range, such as refrigeration temperatures, induce a thermal stress that can affect LAB viability (Sionek et al., 2024), while some LAB strains are cold-tolerant (Liu et al., 2020). Brachkova et al. (2010) also observed that the incorporation of MRS broth in calcium alginate beads containing LAB significantly improved the viability of LAB, maintaining stable cell counts for 6 months of storage at 4 °C. However, temperature poses a significant challenge, as higher storage temperatures (e.g., 30 °C in the present study), while not inducing low-temperature stress, accelerate bacterial metabolic activity, leading to faster nutrient depletion and reduced cell viability. Ghayoomi et al. (2025) entrapped LAB (two *Lactobacillus casei* and *Lactobacillus rhamnosus* strains) in methylcellulose films and sodium caseinate films: they proposed that the LAB strain could live at 4 °C better than at 25 °C during storage for one month, most likely due to faster evaporation of water at ambient temperature (Pereira et al., 2016). According to Ghalehjoogh et al. (2023), the viability of LAB in gelatin/alginate films did not decrease significantly after storage at 4 °C and 25 °C for 14 days; however, the LAB did show a clear downward trend after storage at 37 °C. In another study carried out by Bekhit et al. (2016), a reduction of approximately 50% in the bacterial population was observed when *Lactococcus lactis subsp. lactis* was incorporated into alginate/pectin hydrogels or pure alginate and pectin beads during storage at 30 °C. The bacterial load significantly decreased from 6 log CFU/mg to approximately 3 log CFU/mg after 7 days of storage. In addition to temperature and nutrient availability, factors such as oxygen levels, water activity, and interactions with polar groups in the polymer matrix can also significantly influence bacterial viability during storage, although the exact mechanisms remain not fully understood (Fu and Chen, 2011; Ghayoomi et al., 2025).

### 4.2 Anti-*Listeria* activity of *Lactococcus lactis* LAB3 entrapped in calcium alginate beads supplemented or not with different microbiological culture media conditions

**Figure 2:**
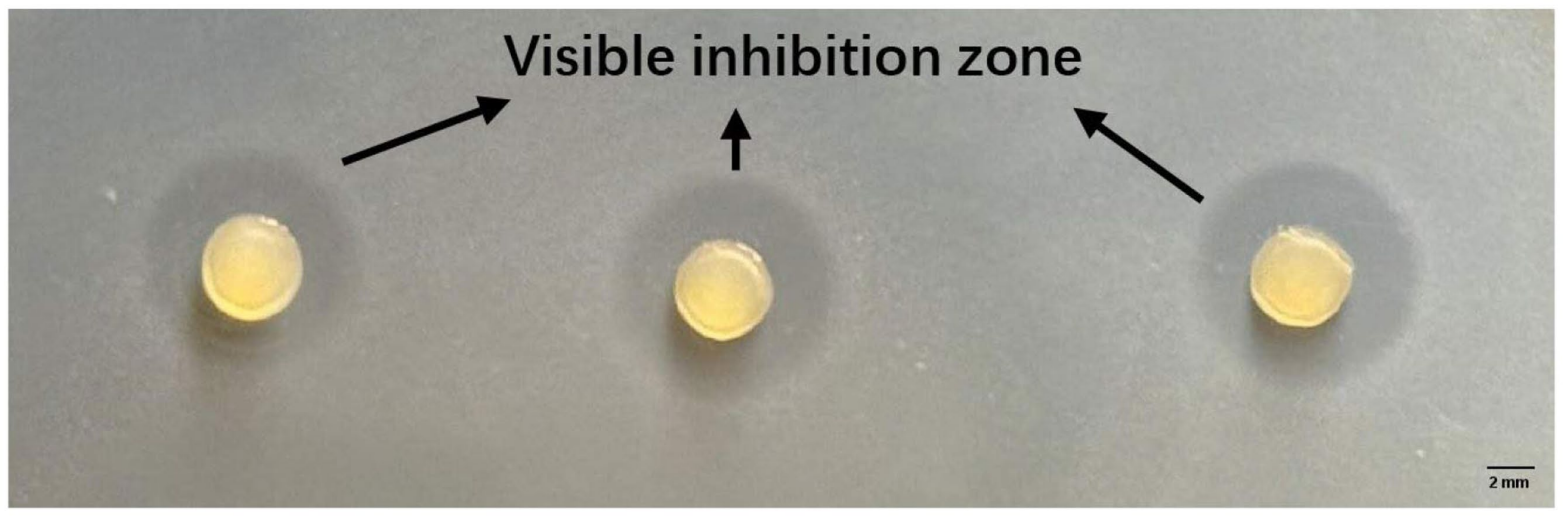
Zones of *Listeria innocua* ATCC 33,090 growth inhibition around Ca-alginate-caseinate beads entrapping *Lc. lactis* LAB3 cells after 1 day of storage in air at 30 °C (agar diffusion assay after 48 h incubation at 30 °C).

The survival of living LAB3 cells in alginate-based beads during long-term storage was a necessary but not a sufficient condition to obtain bioprotective systems: indeed, the entrapped bacteria also must effectively exert their antagonistic activity against *Listeria* spp. bacteria. Antimicrobial activity may be mediated directly by bacterial cells (such as in the case of competition for nutrients), by cell-bound molecules, or by molecules released into the extracellular environment. Production and release of antimicrobial molecules by LAB is known to depend on factors such as substrate composition, cell density, and population kinetics. Therefore, we conducted the classical agar well diffusion method to evaluate the antagonistic activity of *Lc. lactis* LAB3 cells entrapped in polymeric beads. Antimicrobial activity assays against the *L. innocua* ATCC 33090 strain were performed immediately after calcium–alginate bead preparation and after 1-, 5-, 8-, and 12-day storage at 30 °C. As shown in Figure 2, the visible inhibition zone around the beads indicated an effective antagonistic activity against *L. innocua ATCC 33090.* In the absence of microbiological culture medium (Figure 1a), no inhibition zone around calcium–alginate beads entrapping *Lc. lactis* LAB3 cells was detected during storage at 30 °C, while 5.43 ± 0.12 mm and 6.06 ± 1.56 mm inhibition zone diameters around calcium–alginate caseinate beads entrapping *Lc. lactis* LAB3 cells were measured after 0 and 1 day of storage at 30 °C, respectively (Figure 1a). This suggests that caseinate addition in bead formulation stimulates the production of metabolites inhibiting *L. innocua* ATCC 33090 growth. However, in the absence of microbiological growth media, this production of anti-*Listeria* metabolites was not sustained for the 12 days of storage of beads, since the *Listeria* growth inhibition zone was no longer visible from days 2 to 12. Although alginate beads without microbiological culture medium (Figure 1a) showed a better or equivalent viable count of LAB compared to beads supplemented with MRS or M17 during storage for 12 days at 30 °C, their antimicrobial activity decreased rapidly over time. Under these conditions, the highest bacterial counts in beads without medium were reached after 5 days at 30 °C, but no antimicrobial activity was detected. This illustrates the complex relationship between bacterial viability and anti-*Listeria* activity. Although *Lc. lactis* LAB3 cells entrapped in beads without medium maintained high culturability, their ability to produce antimicrobial compounds appeared to be greatly impaired.

Addition of MRS broth (Figure 1b) or M17 broth (Figure 1c) in the formulation of calcium–alginate beads entrapping *Lc. lactis* LAB3 cells resulted in significant *L. innocua* inhibition zones after 0 and 1 day of storage at 30 °C. However, no more inhibition zones could be detected after days 5, 8, and 12. This suggests that nutrient availability within the beads is critical for sustaining *Lc. lactis* LAB3’s anti-*Listeria* effects. Unlike calcium–alginate beads, both MRS and M17 additions to calcium-alginate–caseinate beads resulted in significant inhibition zone diameters over the 12 days of their storage in air. At day 0, beads containing MRS broth exhibited the highest inhibition zones (6.97 ± 1.15 mm), followed closely by beads containing M17 broth (6.85 ± 1.08 mm), while beads without entrapped media displayed a significantly lower inhibition zone (5.43 ± 0.12 mm). This difference indicates that the presence of culture medium within the beads enhances the initial antibacterial activity of LAB3 cells. Nutrient-rich media such as MRS and M17 likely support more robust metabolic activity, enabling higher production of antimicrobial compounds, such as organic acids, bacteriocins, and hydrogen peroxide. In contrast, the lower activity observed for beads without microbiological culture medium suggests that *Lc. lactis* LAB3 cells rely on residual nutrients or intracellular reserves to produce antimicrobial agents. These results also suggest that caseinate addition to alginate with microbiological culture media promotes the production of anti-*Listeria* metabolites by entrapped *Lc. lactis* cells and their release from Ca–alginate–caseinate beads. The diameter of the *L. innocua* inhibition zone after 12 days of storage at 30 °C was significantly higher when Ca–alginate–caseinate beads were supplemented with MRS broth (2.36 ± 0.98 mm) than with M17 broth (1.32 ± 0.74 mm). This is consistent with the previous observation that the culturability of *Lc. lactis* LAB3 cells remained higher after 12 days of storage at 30 °C in beads with MRS broth than in beads with M17 broth. It can thus be concluded that Ca–alginate–caseinate beads supplemented with MRS broth are the best system to both maintain *Lc. lactis* culturability and to promote production and release of anti-*Listeria* metabolites over 12 days of storage at 30 °C in air. Brachkova et al. (2010) also reported that MRS broth promoted the production of antimicrobial metabolites by LAB. Todorov and Dicks (2005) reported that *Lactobacillus plantarum* ST23LD exhibited significantly higher bacteriocin activity (25,600 AU/mL) when grown in MRS broth than in M17 broth, BHI broth, and soy milk (bacteriocin activity ranging between 200 and 800 AU/mL) despite similar growth in these other media. A study on *Lactobacillus plantarum* demonstrated that bacteriocin production is closely linked to nutrient availability. Under nutrient-limited conditions, the synthesis of antimicrobial peptides was significantly reduced, despite sustained bacterial viability (Imade et al., 2021). Across all storage conditions, calcium–alginate–caseinate beads provided better bacterial protection compared to calcium–alginate beads. The antimicrobial activity generally declines over time, but caseinate slows this process, particularly in nutrient-poor environments (i.e., without added medium). The positive effect on anti-*Listeria* metabolite production of caseinate addition to alginate to formulate beads entrapping *Lc. lactis* LAB3 might also result from its buffering capacity. Indeed, LAB exposed to unbuffered environments, such as distilled water, experience stress that can impair their metabolic functions, including the production of antimicrobial compounds. The absence of buffering agents leads to pH fluctuations, adversely affecting metabolic pathways responsible for synthesizing these compounds (Ibrahim et al., 2021). The inclusion of caseinate in polymeric matrices has been shown to enhance the stability and activity of immobilized LAB. Caseinate not only provides structural integrity but also serves as a nutrient source, supporting sustained metabolic activity and antimicrobial production (Savijoki et al., 2006; Léonard et al., 2015).

As shown in Figure 1, the survival rate of LAB cells entrapped in calcium–alginate–caseinate beads was higher, and these beads yielded greater amounts of antimicrobial substances. Notably, the antagonistic activity of the beads was directly linked to the release of antimicrobial compounds from the beads rather than the LAB cells themselves during the storage period. In this context, the improved release of antimicrobial compounds from calcium–alginate–caseinate beads, compared with calcium–alginate beads, could also explain their enhanced anti-*Listeria* activity. Indeed, Léonard et al. (2014) reported a better release of *Lc. lactis* LAB3 cell-free supernatant antimicrobial compounds from sodium alginate–sodium caseinate gels than from sodium alginate gels. These authors also demonstrated that the anti-*Listeria* activity of the *Lc. lactis* LAB3 cell-free supernatant was mainly due to proteinaceous compounds, and partial characterization suggested that *Lc. lactis* LAB3 produces bacteriocin-like substances.

When LAB were effectively incorporated in a sodium caseinate-based coating, the bacteriocins produced by LAB were suggested to maintain the anti-*Listeria* effect for at least 28 days of storage at 10 °C (Giello et al., 2023). In another study by Sánchez-González et al. (2014) regarding films containing LAB based on sodium caseinate and methylcellulose, respectively, it was reported that polysaccharides were more advantageous for producing bacteriocins together with decreased viability of the LAB strain in this matrix. This phenomenon is likely attributed to the polysaccharide environment imposing greater metabolic stress on the microbial cells, as suggested by Gálvez et al. (2007). In the present study, caseinate and alginate concentrations were chosen to obtain an aqueous two-phase system with caseinate droplets dispersed in a continuous alginate phase and a preferential distribution of *Lc. lactis* LAB3 cells in the caseinate phase (Léonard et al., 2013). This morphology of beads likely plays a role in *Lc. lactis* LAB3 culturability but also bacteriocin-like substance production: for instance, class II bacteriocin (such as nisin produced by some *Lc. lactis* strains) production by LAB has been shown to be regulated by cell–cell signaling systems, such as excretion peptides and extracellular accumulation of auto-inducer peptides, including bacteriocin itself. These peptides are believed to permit a quorum-sensing-based regulation of bacteriocin production (Quadri, 2003). In this context, the distribution of *Lc. lactis* LAB3 in caseinate droplets and its absence from the continuous alginate phase might result in a high *Lc. lactis* LAB3 cell density in caseinate droplets, thereby promoting bacteriocin-like substance production. Therefore, calcium–alginate–caseinate beads were inoculated with *Lc. lactis* LAB3 cells at two different levels to investigate the effect of inoculum size on their culturability and its anti-*Listeria* activity over 12 days of storage at 30 °C in closed bottles.

### 4.3 Effect of initial inoculum load on anti-*Listeria* activity of *Lactococcus lactis* LAB3 entrapped in calcium–alginate–caseinate beads

Medium composition and the incorporation matrix greatly affected the viability and anti-*Listeria* activity of LAB3 cells in alginate-based beads. Nutrient-rich media (MRS and M17) supported long-term and higher antibacterial activities of LAB entrapped in calcium–alginate–caseinate beads by enhancing antimicrobial metabolite (e.g., bacteriocin) production, while beads lacking an added microbiological culture medium showed reduced activity despite high cell counts. Since our goal is to obtain a long-term storage system that can be adopted in real food products, the optimal parameters supporting antagonistic activity should be taken into consideration.

**Figure 3:**
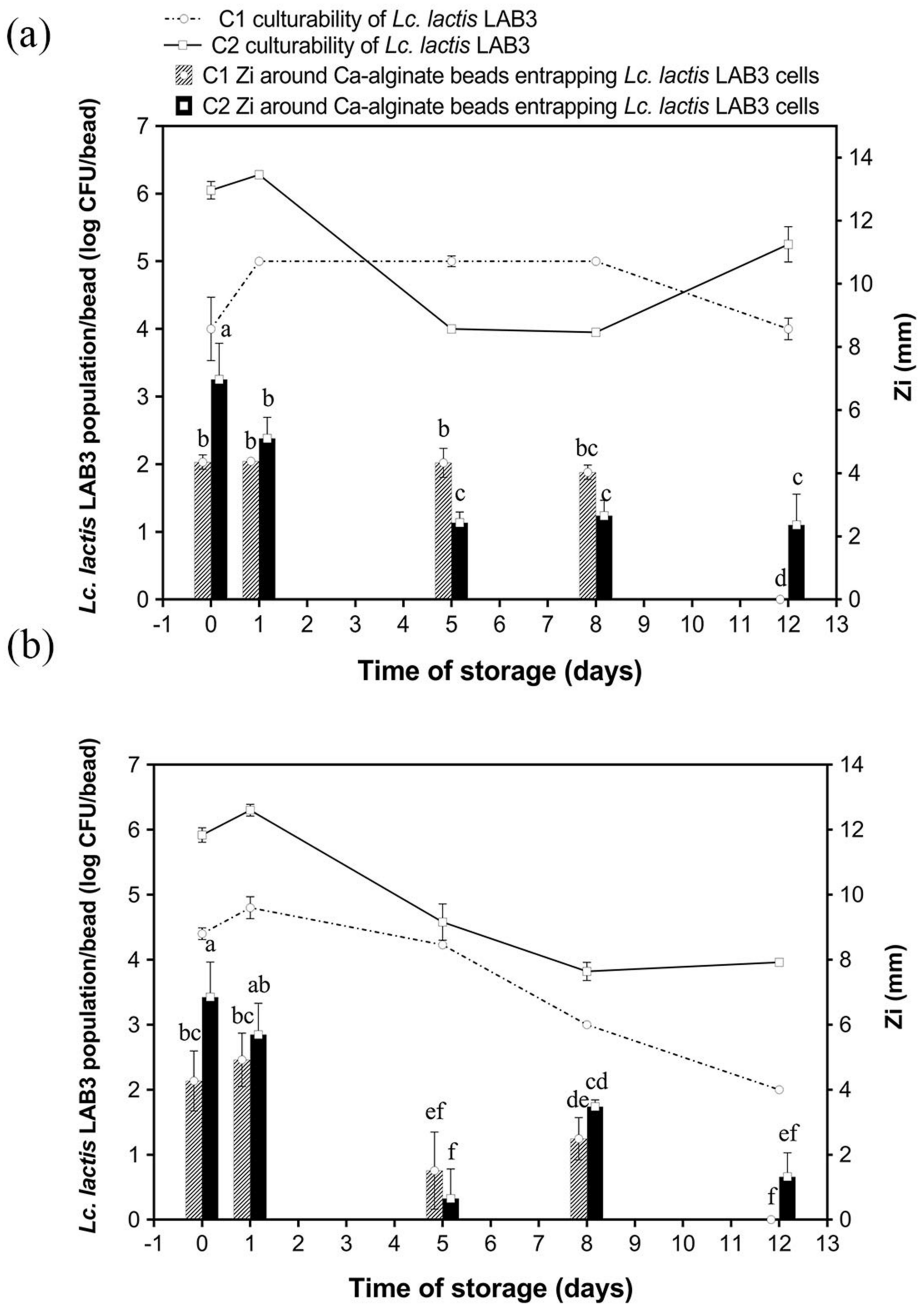
A culturability and a anti-*Listeria* activity of *Lactococcus lactis* LAB3 entrapped in Ca-alginate-caseinate beads storage in air at 30 °C over 12 days. Biopolymeric matrices compositions of beads: **(a)** 1.5% (w/w) sodium alginate + 4% (w/w) sodium caseinate +20% (w/w) MRS broth; **(b)** 1.5% (w/w) sodium alginate + 4% (w/w) sodium caseinate + 20% (w/w) M17 broth. Initial inoculum load of *Lc. lactis* LAB3 cells in initial liquid biopolymeric matrices before beads preparation: (C1) ~10^6^ CFU mL^−1^, (C2) ~10^8^ CFU mL^−1^. All beads were incubated in sealed bottles. The culturability of LAB cells in Ca-alginate-caseinate bead (displayed by reduction in log CFU per bead) was determined using classical enumeration on agar, represented by the lines. The antibacterial activity (Zi) was estimated by measuring the diameters of *Listeria innocua* 33,090 growth inhibition zones around Ca-alginate-caseinate bead placed on the surface of agar plates, shown by bars. The inoculum concentration of *L. innocua* 33,090 used for the antimicrobial activity (Zi) assay was 5 × 10^5^ CFU mL^−1^. Data represents the mean ± SD of three independent experiments. Different letters above bars indicate significant differences (one-way ANOVA with LSD post hoc, *p* < 0.05) in each figure. No inhibition zone of beads without cells entrapped was detected.

The initial LAB inoculum size stands as another crucial parameter to investigate, as studies have shown that higher inoculum cell densities enhance initial antibacterial activity through increased bacteriocin production, though they may also accelerate nutrient depletion and reduce long-term efficacy (Brachkova et al., 2010; Léonard et al., 2015). Therefore, calcium–alginate–caseinate beads containing MRS and M17 broth were tested to evaluate the effect of initial inoculum size on antimicrobial activity over time. Anti-*Listeria* activity and *Lc. lactis* LAB3 culturability of calcium–alginate–caseinate beads with MRS broth (Figure 3a) or M17 broth (Figure 3b) inoculated with either 10^6^ CFU mL^−1^ (C1) or 10^8^ CFU mL^−1^ (C2) of *Lc. lactis* LAB3 were monitored for 12 days of storage at 30 °C in closed bottles. When beads were added to MRS broth (Figure 3a), the initial inoculum load of *Lc. lactis* LAB3 cells resulted in an initial cell density of approximately 4 log CFU/bead for C1 and 6 log CFU/bead for C2, respectively. Accordingly, C2 initial inoculum load yielded higher initial antimicrobial activity (inhibition zone diameters of 6.97 ± 1.15 mm and 4.35 ± 0.23 mm for C2 and C1 inoculum size, respectively). As mentioned previously, higher bacterial loads often stimulate quorum-sensing mechanisms, enhancing the synthesis of antimicrobial metabolites due to cell-to-cell communication (Zeng et al., 2023). Such mechanisms might thus explain the large inhibition zone diameters for beads with the highest inoculum size (C2); higher cell density was thus associated with a higher anti-*Listeria* activity, likely due to higher bacteriocin-like substance production by *Lc. lactis* LAB3.

With a lower inoculum size (C1), the anti-*Listeria* activity of the calcium–alginate–caseinate beads stored at 30 °C was maintained for 8 days, with no significant variation in the inhibition zone diameters (approximately 4 mm) (Figure 3a). This stability of anti-*Listeria* activity over time was attributed to the increase in cell number by 1 log CFU/bead after 1 day, stabilizing around 5 log CFU/bead during the subsequent period, and maintaining this level until “Day 8.” However, the *Listeria* growth inhibition zone was no longer detected after 12 days: this might be due to a too low *Lc. lactis* LAB3 cell density and/or metabolic activity in the beads.

For the highest size inoculum of C2, the diameters of inhibition zones around beads gradually decreased with increasing storage time at 30 °C; however, *L. innocua* 33,090 inhibition remained effective over the days of storage at 30 °C (i.e., a 2.5–3.0 mm inhibition zone diameter around beads was recorded at days 5 and 12), unlike when beads were inoculated with a lower inoculum size. Following inoculation of beads, the evolution of anti-*Listeria* activity over time is consistent with the corresponding *Lc. lactis* LAB3 populations. After 8 days of incubation, the cell density decreased from 6.28 + 0.04 on D1 to 3.95 + 0.04 log CFU/bead on D5, while the cell density remained at ~5 CFU/bead from D1 to D8 in the C1 case (Figure 3a). It can be concluded from these results that the long-term antimicrobial efficacy depends on inoculum size: A higher initial inoculum size (C2) supports sustained anti-*Listeria* activity over an extended period (12 days), with inhibition zone diameters exceeding 2 mm. However, high inoculum size leads to a rapid decline in anti-*Listeria* activity: the inhibition zones decreased after 1 day, correlating with a rapid decline in cell density after a maximum of ~4 log CFU/bead. Lower inoculum size (C1) leads to less rapid decline: it results in more consistent inhibition (~4 mm *L. innocua* growth inhibition zone diameter) for 8 days, but it failed to maintain anti-*Listeria* activity on day 12. This stability for the first 8 days is attributed to a steady bacterial population that increases on the first day and remains stable (~5 log CFU/bead). In the study conducted by Bevilacqua et al. (2010), when *Lactobacillus plantarum* was loaded into alginate–caseinate gels with a higher initial inoculum load (about 10^9^ CFU/g), the culturability remained at 10^5^ CFU/g after 12 days of storage at 30 °C. As noted by Ünlü et al. (2015), who monitored *L. sakei* CTC strain 494 growth in MRS medium at 30 °C, rapid decline of growth rate over time cannot be ascribed to the self-inhibition phenomenon caused by LAB caused by lactic acid production, since the inhibitory effect of lactic acid production does not fully explain the growth inhibition, especially at pH 6.5, where 99.8% of the lactic acid was dissociated, while the microbial growth inhibitory properties of lactic acid have been shown to owe it to its undissociated form (Arrioja-Bretón et al., 2020). The growth reduction of *L. sakei* CTC 494 in MRS broth is rather due to the exhaustion of nutrients (likely a vitamin, an amino acid, or a peptide), as evidenced by the increased final biomass yield as well as the production of lactic acid due to the longer-term availability of nutrients (Ünlü et al., 2015). In addition, in our study, the bacteriocin-like substances accumulated on “Day 0” likely trigger a self-regulatory mechanism in the production of *Lc. lactis* LAB3. Due to limited immunity to their own bacteriocin-like substances, elevated extracellular concentrations likely inhibit further production (Leroy and De Vuyst, 2001). This regulation ensures that bacteriocin levels remain within a functional range, maintaining antimicrobial activity while preventing toxicity to the producer cells.

Similar results were observed for LAB3 in calcium–alginate–caseinate-beads containing M17 medium (C2 inoculum), as seen in Figure 3b. A substantial inhibition zone diameter (~6 mm) on both “Day 0” and “Day 1” declined rapidly after “Day 1,” reaching ~1 mm by “Day 12.” This is consistent with higher initial culturability (~6 log CFU/bead), which declined steeply after “Day 1,” reaching ~4 log CFU/bead by “Day 12.” With a lower inoculum size (C1), the culturability of *Lc. lactis* LAB3 cells and their anti-*Listeria* activity, as estimated by the growth inhibition zone diameter, were both lower in M17 broth than in MRS broth. This may be due to a greater limitation of nutrients available for sustained growth in M17 broth. Indeed, MRS broth contains approximately 20.0 g/L of nitrogen sources (peptone and autolytic yeast extract), while M17 broth provides 17.5 g/L from a combination of tryptone, peptic digest of meat, papaic digest of soybean meal, yeast extract, and meat extract. The slightly higher nitrogen content of MRS broth may account for its greater effectiveness in maintaining *Lc. lactis* LAB3 culturability over 12 days and in sustaining long-term antimicrobial activity.

### 4.4 The effect of modified atmospheres on anti-*Listeria* activity of *Lactococcus lactis* LAB3 entrapped in calcium–alginate–caseinate beads

The shelf life of refrigerated perishable food products is influenced by factors such as gas composition in modified atmosphere packaging (MAP), storage temperature, initial microbial load, and the presence of additives (McMillin, 2008). Indeed, MAP is a widely used technology to extend shelf life and/or enhance the microbial safety of refrigerated perishable foods, whereas biopreservation remains at an early stage of commercial application. In this context, beads entrapping bioprotective LAB, such as *Lc. lactis* LAB3, would be of practical interest only if they further extend shelf life and/or improve the microbial safety of foods stored under MAP conditions. However, combining MAP with bioprotective LAB applications, such as *Lc. lactis* LAB3 entrapped in calcium–alginate–caseinate beads, is advantageous only if the anti-*Listeria* activity of *Lc. lactis* is not impaired by storage in a modified atmosphere.

**Figure 4:**
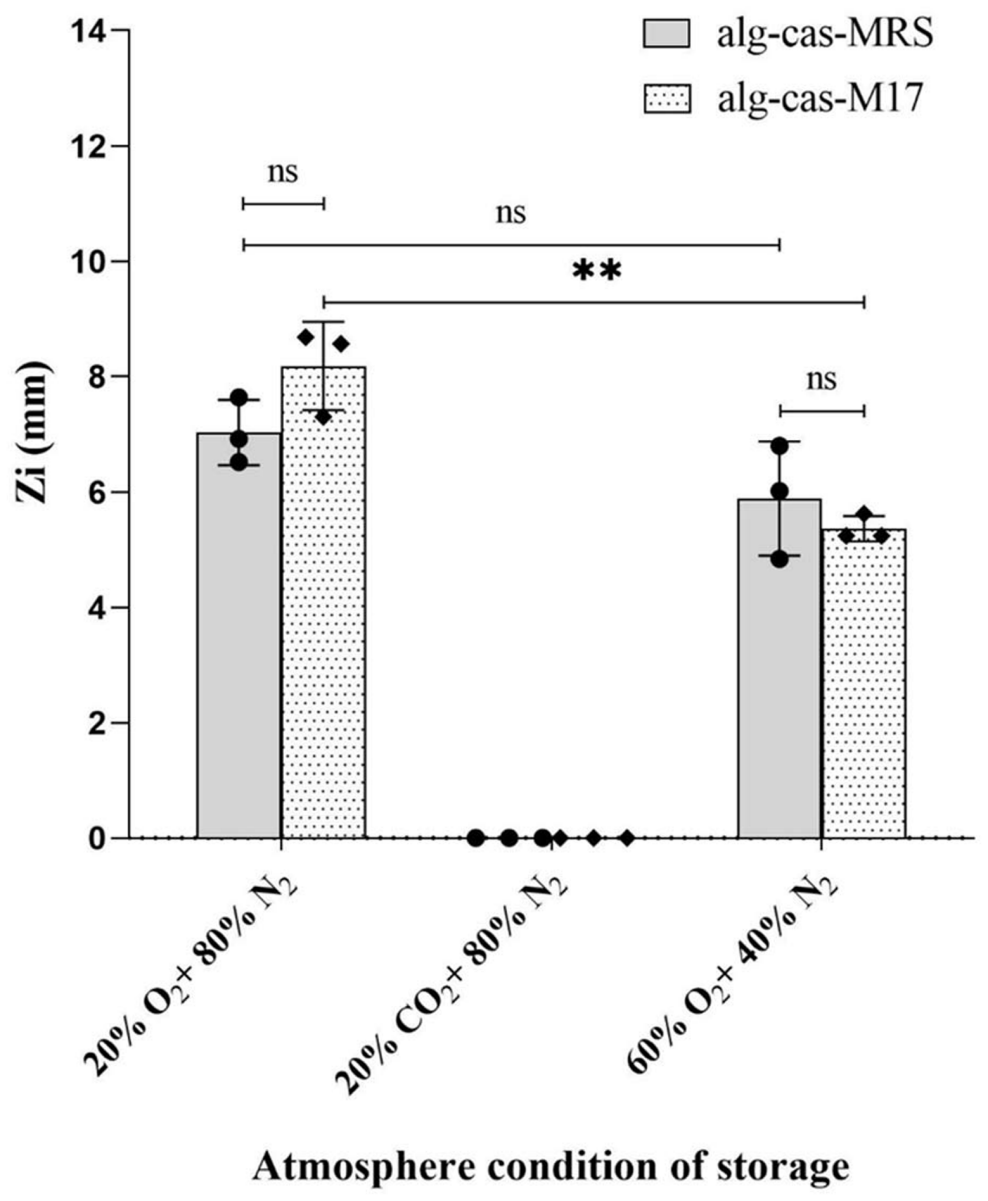
Effect of modified atmospheres on the antimicrobial activity of *Lactococcus lactis* LAB3 entrapped in Ca-alginate-caseinate bead storage at 30 °C (closed in cylinders for 4 days). Inhibition zone diameters were measured by agar well diffusion method (*n* = 3) Ca-alg-cas-MRS: 1.5% (w/w) sodium alginate 4% (w/w) sodium caseinate, supplemented with 20% (w/w) MRS broth; Ca-cas-M17: 1.5% (w/w) sodium alginate + 4% (w/w) sodium caseinate, supplemented with 20% (w/w) M17 broth. The diameters of beads are 2.79 ± 0.07 mm and 2.79 ± 0.1 mm, respectively (*n* = 15) Initial inoculum load of *Lc. lactis* LAB3 cells in initial liquid biopolymeric matrices before beads preparation: 10^8–9^ CFU mL^−1^. The antibacterial activity (Zi) was estimated by measuring the diameters of *Listeria innocua* 33,090 growth inhibition zones around Ca-alginate-caseinate bead placed on the surface of agar plates. The inoculum concentration of *L. innocua* ATCC 33,090 used for the antimicrobial activity (Zi) assay was 5 × 10^5^ CFU mL^−1^. Data represents mean values ± SD. Different capital letters report statistically significant (*p* < 0.01, *n* = 3).

To assess this, the antagonistic activity of calcium–alginate–caseinate beads containing *Lc. lactis* LAB3 against *L. innocua* ATCC 33090 was evaluated after 4 days of storage in one of the following atmospheres: 80% (v/v) N_2_ and 20% (v/v) O_2_ (mimicking air composition), 80% (v/v) N_2_ and 20% (v/v) CO_2_ (mimicking high-CO_2_ MAP), or 60% (v/v) O_2_ and 40% (v/v) N_2_ (mimicking high-oxygen MAP, or HOMAP), which is commonly used to preserve the bright red color of oxymyoglobin in raw bovine meat (Figure 4). The bead diameters—calcium–alginate–caseinate–MRS (2.79 ± 0.07 mm) and calcium–alginate–caseinate–M17 (2.79 ± 0.10 mm)—showed no statistically significant difference between the two groups (*p* < 0.05). The initial inoculum load on day 0 was ~6 log CFU/bead for both samples (Table 2). Control beads without *Lc. lactis* LAB3 cells produced no inhibition zones (data not shown), confirming that all observed antimicrobial activities in this experiment were entirely due to the antagonistic activity of *Lc. lactis* LAB3 against *L. innocua* 33,090.

After 4 days of storage at 30 °C in a 20% (v/v) O_2_ atmosphere, Zi values of 7.03 ± 0.46 mm and 8.18 ± 0.63 mm were recorded for calcium–alginate–caseinate-MRS beads and calcium–alginate–caseinate-M17 beads, respectively. These values are comparable to those obtained for D0 beads inoculated in closed bottles with air after 48 h, which measured 6.9 ± 1.15 mm and 6.85 ± 1.08 mm for alginate–caseinate–MRS beads and alginate–caseinate–M17 beads, respectively (Figures 3a,b).

Observation of Figure 4 reveals that *L. innocua* growth inhibition zone diameters were slightly smaller after 4 days of incubation at 30 °C in a HOMAP atmosphere (Zi values of 5.89 ± 0.81 mm and 5.37 ± 0.18 mm for calcium–alginate–caseinate-MRS and calcium–alginate–caseinate-M17 beads, respectively) compared with incubation in a 20% (v/v) O_2_–80% (v/v) N_2_ atmosphere (Zi values of 7.03 ± 0.46 mm and 8.18 ± 0.63 mm, respectively). However, antagonistic activity against *L. innocua* ATCC 33090 was lost after 4 days of incubation in a 20% (v/v) CO_2_–80% (v/v) N_2_ atmosphere. The antimicrobial activity of *Lc. lactis* LAB3 in the presence of 20% (v/v) and 60% (v/v) O_2_ is consistent with its facultative anaerobic character. Amanatidou et al. (1999) monitored the growth at 8 °C of another *Lc. lactis* strain on an agar-surface model under various high-oxygen and high-carbon dioxide atmospheres. A 90% (v/v) O_2_ atmosphere reduced the growth rate and extended the lag phase, while a 10% (v/v) CO_2_ atmosphere did not modify either the growth rate or the lag phase duration compared with a 20% (v/v) O_2_ atmosphere. In the present study, the incubation temperature (30 °C) and the *Lc. lactis* strain used differed; only anti-*Listeria* activity (assessed by agar diffusion assay) was measured, not culturability. Moreover, a higher CO_2_ proportion (20% (v/v)) and lower O_2_ proportion were used, making direct comparisons impossible. The significant *L. innocua* ATCC growth inhibition diameter measured after 4 days of storage of beads in a 60% (v/v) O_2_ atmosphere indicates significant production of bacteriocin-like substances by *Lc. lactis* LAB3. Oxygen supports the growth of bacteria, including spoilage organisms such as *Pseudomonas* and LAB, which can produce slime, sourness, and/or off-flavors when reacting 10^7–8^ CFU/g (Franke et al., 2017). However, van Niel et al. (2002) reported that *Lc. lactis* subsp. *lactis* ATCC 19435 grows at a slower rate in the presence of oxygen, with reduced activity in oxygen-rich conditions attributed to oxidative stress impairing bacterial viability. Therefore, storage for 4 days at 30 °C in a 60% (v/v) O_2_ atmosphere might have reduced the viability of *Lc. lactis* LAB3. Conversely, Cabo et al. (2001) reported that nisin production increased with higher oxygen saturation, suggesting that 60% O₂ may be optimal for the production of nisin Z. This indicates that nisin biosynthesis may be associated with an oxidative metabolic pathway.

The antimicrobial activity of CO₂ is influenced by multiple factors, such as concentration, partial pressure, temperature, pH, water activity, the type of microorganism, and growth stage. Literature suggests that an initial headspace concentration of at least 20% CO₂ is generally required for bacterial inhibition (Meredith et al., 2014). The absence of inhibition zones following 4 days of storage in a 20% (v/v) CO_2_ atmosphere warrants further investigation to determine whether carbon dioxide inhibits *Lc. lactis* LAB3 growth and/or bacteriocin-like substance production. As noted by Kolbeck et al. (2020), research on CO_2’s_ effects on bacterial metabolism remains limited compared with studies on its bacteriostatic action.

## 5 Conclusion

The study investigated the entrapment of *Lc. lactis* LAB3 cells within calcium–alginate–caseinate and calcium–alginate beads, supplemented or not with either MRS or M17 broth. Monitoring these beads over 12 days of storage at 30 °C showed that calcium–alginate–caseinate beads were more effective than calcium–alginate beads in maintaining both the culturability of entrapped *Lc. lactis* LAB3 cells and their anti-*Listeria* activity over time. Moreover, the addition of 20% (w/w) MRS or M17 broth to the bead formulation improved *Lc. lactis* LAB3 culturability and anti-*Listeria* activity, with MRS broth yielding better results. The higher anti-*Listeria* activity was attributed to the enhanced production of bacteriocin-like substances by entrapped *Lc. lactis* LAB3 cells. Calcium–alginate–caseinate beads containing MRS broth proved effective in preserving viable bioprotective LAB even under simulated cold chain break conditions. Inoculation of beads at 10^8^ CFU mL^−1^ improved the stability of *Lc. lactis* LAB3 culturability and anti-*Listeria* activity over time compared to inoculation at 10^6^ CFU mL^−1^. Finally, the evaluation of anti-*Listeria* activity after 4 days of storage at 30 °C in different atmospheres—20% (v/v) O_2_–80% (v/v) N_2_ (mimicking air), 60% (v/v) O_2_–40% (v/v) N_2_ (mimicking high-oxygen modified atmosphere packaging, HOMAP), and 20% (v/v) CO_2_–80% (v/v) N_2_ (to favor the bacteriostatic effect of CO_2_)—revealed that while the anti-*Listeria* activity of beads was maintained in atmospheres containing up to 60% (v/v) O_2_ but was impaired in the presence of 20% (v/v) CO_2_. HOMAP can be combined with entrapped *Lc. lactis* LAB3 as a bioprotective strategy to extend shelf life and/or improve the microbiological safety of perishable foods, whereas a 20% (v/v) CO_2_–80% (v/v) N_2_ atmosphere would not be beneficial. Further research is required to elucidate the mechanisms by which CO_2_ impairs bacteriocin-like substance production by entrapped *Lc. lactis* LAB3 cells.

## Data Availability

The raw data supporting the conclusions of this article will be made available by the authors, without undue reservation.
